# Magnetic‐Field‐Tuned Spin‐Dependent D‐Band and Bifunctionality in L1_0_‐FeCoPt for Enhanced Methanol Oxidation

**DOI:** 10.1002/advs.78112

**Published:** 2026-09-30

**Authors:** Qiqi Mo, Kaijie Liu, Ruixi Liu, Yong Juan, Sihan Wang, Jialong Liu, Wei Wang

**Affiliations:** ^1^ Department of Physics and Electronics School of Mathematics and Physics Beijing University of Chemical Technology Beijing China

**Keywords:** d‐band, FeCoPt, magnetic field, methanol oxidation reaction, spin

## Abstract

The spin‐dependent electronic structure of transition‐metal alloys plays a key role in tuning the magnetic‐field‐responsive behavior, which plays a critical role in enhancing the electrocatalytic properties under an external magnetic field. This work presents a facile hydrothermal synthesis of L1_0_‐phase FeCoPt nanoparticles with tunable elemental compositions. The relationship between MOR mass activity and Co content illustrates a volcano shape, and sample FeCoPt‐4 possesses the best activity in acidic media, 7.16 times higher than that of commercial Pt/C. In addition to composition, methanol oxidation reaction (MOR) performance can be further enhanced by varying methanol concentration (1 to 10 m) and applying an 8000 Oe magnetic field. First‐principles calculations reveal that the d‐band center of Pt 5d orbitals and the net spins of Fe/Co can be tuned with increasing Co content, resulting in optimized magnetic field response and enhanced electrocatalytic properties. The combination of experimental and theoretical analysis shows that the MOR process is kinetically governed by the reaction of CO* and OH*, which is affected by the Co content, methanol concentration and acidic/alkaline solution. This research clarifies the multi‐factor enhancement mechanism by leveraging spin‐dependent electronic structure and bifunctional effect, enabling a novel approach for designing high‐performance electrocatalysts for direct methanol fuel cells.

## Graphical abstract

The d‐band center and bifunctional mechanism jointly regulate the amounts of adsorbed OH* and CO*, thereby enhancing electrocatalytic performance, while the application of an external magnetic field further boosts this performance.

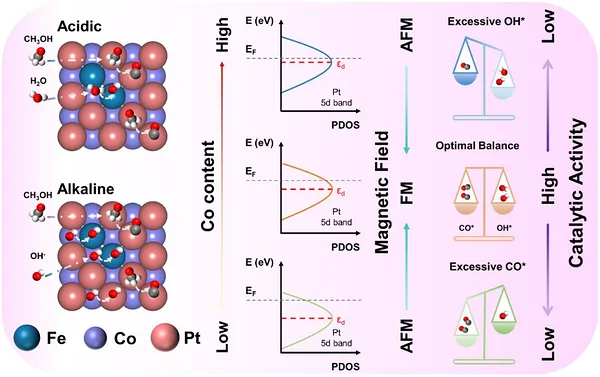

## 1 Introduction

Direct methanol fuel cells (DMFCs) are an attractive and potential energy transformation system due to their ease of fuel storage and transport [1, 2]. Because the sluggish kinetics of the methanol oxidation reaction (MOR) at the anode limit the overall performance, various novel Pt‐based nanomaterials are developed to overcome the CO poisoning drawbacks, thus increasing the activity and stability to meet the commercial standard [3, 4, 5]. The MOR process involves a six‐electron transfer from CH_3_OH to the final CO_2_ product, with the reaction between CO* and OH* typically serving as the rate‐determining step. According to the Sabatier principle, a fundamental strategy for improving intrinsic MOR activity is to optimize the adsorption/desorption thermodynamics of key intermediates [6, 7]. Therefore, alloying Pt with transition metal elements (M = Fe, Co, Ni) represents a highly promising approach. Strengthening the synergistic effect within these alloys plays a crucial role in optimizing the reaction kinetics and achieving superior MOR performance.

Two reactions mechanisms are raised to explain the synergistic effect and design novel alloy electrocatalysts, namely d‐band center and bifunctional mechanism. The d‐band model uses a single energy level ε_d_ (the d‐band center) to approximate interacting d‐states, which corresponds to the narrow d‐band limit of the Newns‐Anderson model [8, 9, 10]. According to this model, variations in adsorption energy on transition metal (TM) surfaces correlate with the shift of the d‐band center relative to the Fermi energy. A large upward shift suggests the formation of more empty anti‐bonding states to bind the adsorbate. Alloying Pt with transition metals can effectively modulate the d‐band center of Pt element, thereby altering the binding strength of carbon/oxygen species and finally promoting the MOR performance [11, 12]. On the other hand, the bifunctional mechanism considers the combined functionalities of two different active sites [13]. The CO poisoning phenomenon deteriorates the catalyst stability. The adsorbed CO on Pt can be oxidized into CO_2_ by using the co‐adsorbed hydroxyl racial on the neighbor metal sites such as Ru, Co and Fe [14, 15]. The combination of both mechanisms is beneficial to enhancing the activity and selectivity [16]. Nevertheless, the performance enhancement is limited and requires novel physical principles to tune the electrocatalytic process.

Traditional electrochemical and theoretical studies have long focused on the charge properties of electrons, while generally neglecting the role of spin in electrochemical reaction processes. Recently, spin electrochemistry, especially magnetic field‐assisted electrocatalysis, has emerged as an effective methodology for enhancing electrochemical reactions. As an external stimulus, a magnetic field can leverage various effects—such as the magnetothermal, magnetohydrodynamic (MHD), Maxwell stress, Kelvin force, and spin‐selective effects—to augment the electrocatalytic performance [17, 18, 19, 20, 21, 22]. The spin polarization induced by an external magnetic field governs the spin states of intermediate species, which is closely tied to the spin‐dependent electronic structure and magnetic field response of the spin catalyst. For Cr_2_Te_3_/CrOOH heterostructures with a paramagnetic/antiferromagnetic configuration and ferromagnetic CoFe_2_O_4_, the external magnetic field promotes the conversion of singlet reactants (e.g., OH^−^ or H_2_O) to triplet oxygen molecules (O_2_), which optimize the electron transfer between adsorbed intermediates and active sites in the oxygen evolution reaction (OER) [23, 24]. The magnetization of cobalt nanoparticles promotes electron transitions to higher spin states, thereby increasing the number of unpaired 3d electrons. Given that spin‐induced orbital interactions alter electron energy levels [25], this change of spin‐dependent electronic structure enhances the spin magnetic moment, further lowering the activation energy for electron transfer and consequently boosting the oxygen reduction reaction (ORR) [26]. Therefore, magnetic fields can precisely control spin orientation and spin‐dependent electronic structures, thereby optimizing the adsorption state of intermediates and lowering the reaction energy barrier [3].

Ternary magnetic Pt‐based alloys have extra regulatory factors to tune the structure ordering degree, coordination environment and spin distribution [27]. The spin‐polarized alloys possess different spin‐up and spin‐down electronic structure, leading to net spins. Even with a small amount of ternary alloy, the density of states for spin‐up and spin‐down are enlarged, which can further increase the magnetic field response [28]. However, the previously used metals such as Mn, Bi and Cr can only be alloyed a little due to their big difference of electronegativity compared to Pt [29]. The continuous regulation of spin‐dependent structure demands the complete miscibility among all the metal components. The importance of this factor to magnetic field response in MOR process is seldom reported.

Herein, we propose a facile design for continuously controlling the electronic structure and d‐band center of FeCoPt alloys by alloying various amounts of Co into FePt nanocatalysts. The electronic and spin properties of the FeCoPt alloy is effectively adjusted due to the miscibility among Fe, Co and Pt. The activity and magnetic field response of FeCoPt are evaluated via cyclic voltammogram (CV) testing in acidic and alkaline methanol solution. The methanol concentrations are varied as well to study the change of adsorbate under magnetic fields. First‐principles calculations are applied to analyze the effects of Co content on the net spin, electronic structure, d‐band center and reaction barrier during MOR process, given the way to tune the spin‐dependent electronic structure as well as MOR performance.

## 2 Results and Discussion

The morphologies and microstructures of the pristine FeCoPt samples before annealing were characterized by using transmission electron microscopy (TEM). As shown in Figure S1a, the uniformly distributed nanoflowers with size of about 20 nm are FeCoPt alloys. The transparent substance is C substrate, and FeCoPt is adsorbed on it via ultrasonication. The selected area electron diffraction patterns (SAED) in Figure S1b show distinct diffraction rings, which are attributed to (111), (200), (220) and (311) planes in face‐center‐cubic (FCC) phase, respectively. The high‐resolution TEM (HRTEM) images in Figure S1c,d demonstrate that the nanoflower is composed of many nanograins with rich grain boundaries. The crystal lattice with a spacing of 0.22 nm can be attributed to the (111) planes.

The annealing process promotes the nanoflowers melted and fused into larger nanoparticles (Figure S2b_1_). The acquired SAED patterns for FeCoPt‐2 are alike to those of pristine FeCoPt/C, except that two extra distinct diffraction rings appear. All the diffraction patterns can be attributed to (001), (110), (111), (200), (220) and (311) planes in L1_0_ phase, respectively (Figure S2b_2_). Despite of phase transition, the annealing process improves the crystallinity, as the orientations of adjacent nanoparticles are approaching and the lattice fringes go through an individual nanoparticle (Figure S2b_3_). The two‐dimensional lattice fringes with perpendicular atomic planes are attributed as (200) and (001) planes, respectively. Moreover, Figure 1a,b exhibits high‐resolution images recorded by the aberration‐corrected high‐angle annular dark field scanning transmission electron microscopy (HAADF‐STEM). The Fast Fourier Transformation (FFT) pattern and the distinct lattice fringes illustrate typical (110) and (001) crystal planes. Intensity profile for the while box displays the ordered atomic arrangement with alternating Pt‐rich (bright) and Fe/Co‐rich (dim) atomic columns, further confirming its L1_0_‐phase structure (Figure 1c) [30]. For FeCoPt‐2, the actual element ratio of Fe:Co:Pt is 23:25:52 determined by inductively coupling plasma‐mass spectroscopy (ICP‐MS). The precursor ratio and actual ratio of Fe:Co:Pt for all five samples are illustrated in Table 1. The EDS measurements provide a consistent trend as ICP‐MS results. Varying the amount of the precursor Co(acac)_2_ can change the iron, cobalt and platinum composition of FeCoPt samples, but the morphology shows almost no variation (Figure S2). Meanwhile, the lattice fringes can be indexed as the (110), (111) and (001) planes of L1_0_ phase.

**FIGURE 1:**
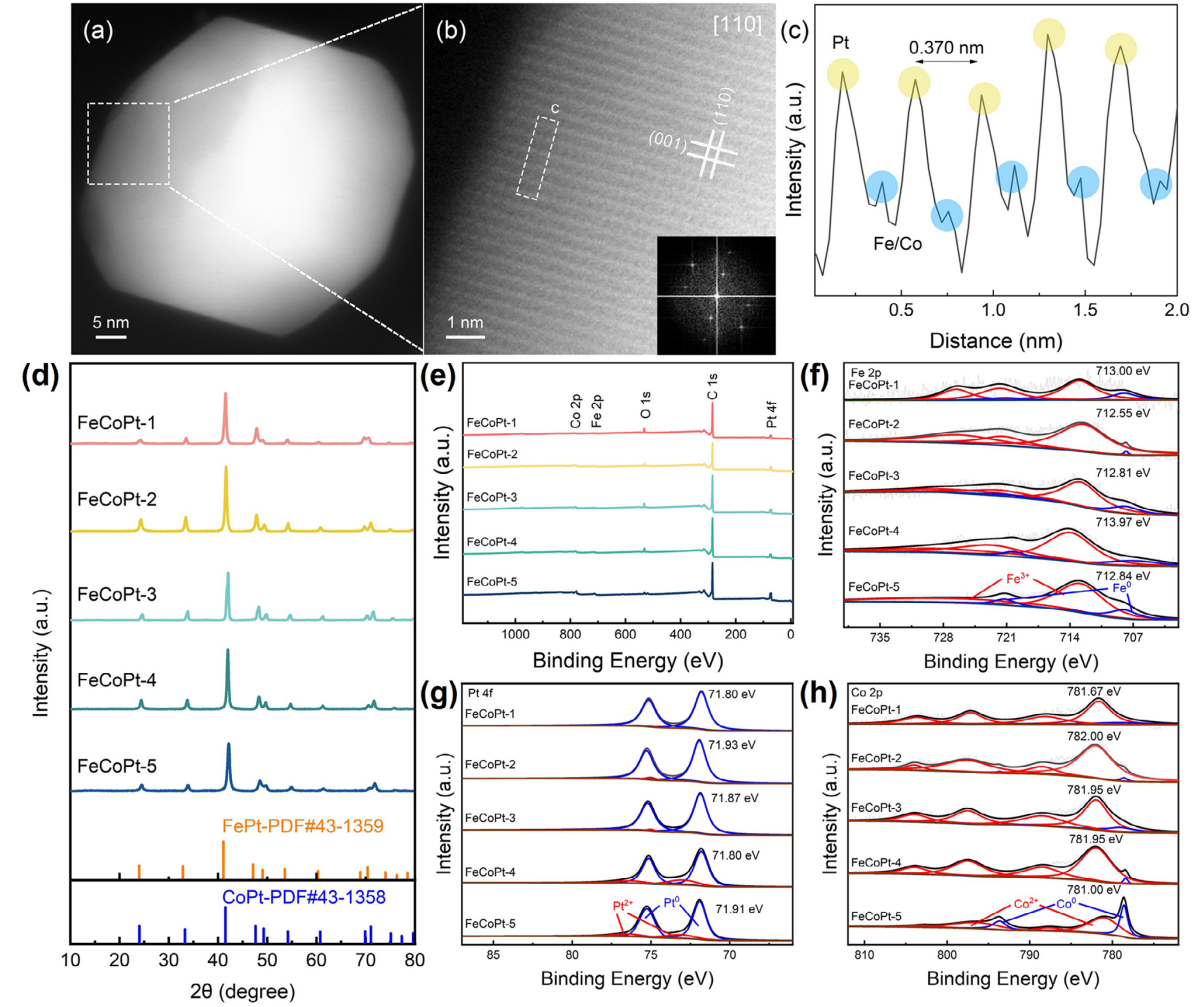
(a,b) HAADF‐STEM images along the [110] zone axis, showing distinct (001) and (110) planes of L1_0_ structure. Inset is the FFT pattern. (c) Z‐contrast intensity profile from the white box in (b). The crystal structure and electronic structure of five FeCoPt catalysts with different compositions. (d) XRD patterns. (e) XPS survey spectra. High‐resolution XPS spectra for (f) Fe 2p; (g) Pt 4f and (h) Co 2p, respectively.

**TABLE 1:** Precursor atomic ratios and actual atomic ratios measured by ICP‐MS and EDS.

Sample	Precursor	ICP‐MS	EDS
	Fe:Co:Pt	Fe:Co:Pt	Fe:Co:Pt
FeCoPt‐1	1:1:3	22:18:60	24:21:55
FeCoPt‐2	1:2:3	23:25:52	21:31:48
FeCoPt‐3	1:3:3	16:38:46	17:37:46
FeCoPt‐4	1:4:3	15:43:42	17:42:41
FeCoPt‐5	1:5:3	12:53:35	17:45:38

The crystal structure was investigated by XRD. All the diffraction patterns fit well with the PDF cards for L1_0_‐FePt (PDF#45‐1359), and the peaks located at 24.2°, 33.5°, 40.3°, 46.9°, 68.4° can be indexed as (001), (110), (111), (200) and (111) planes, respectively (Figure 1d). Since the atomic radius of the incorporated cobalt atom is smaller than those of Fe, and Pt, the (111) diffraction peaks of the FeCoPt nanocatalysts move to larger angles with increasing Co contents, indicating the successful alloying (Figure S3).

The XPS characterization further confirms the formation of FeCoPt alloy samples (Figure 1e–h). Survey spectra shown in Figure 1e indicates the coexistence of Fe, Pt, Co and C elements in the five samples. Regarding FeCoPt‐2, the Fe 2p_3/2_ spectrum can be fitted into metallic state Fe^0^ at 707.79 eV and oxidation state Fe^3+^ at 712.55 eV. This peak position is the lowest compared to the other samples. Moreover, the Pt 4f spectrum can be attributed to Pt 4f_7/2_ at 71.93 eV and Pt 4f_5/2_ at 75.28 eV, but this peak position is the highest among all the samples. When changing the Co content, the peak shifts to a lower binding energy. Similarly, the Co spectra can be fitted to different valence states (Co^0^/Co^2+^). The 2p_3/2_ peak of Co^2+^ is ranging from 781.00 to 782.00 eV, among which the peak position for FeCoPt‐2 is the highest. It is known that the peak shifting toward lower energy means gaining electrons from adjacent atoms [31]. Herein, the electrons belonging to Pt and Co atoms are inclined to transfer to Fe atoms for the FeCoPt‐2 sample. Among the five samples, FeCoPt‐4 is also special owing to its lowest Pt peak position, which demonstrates that Pt atoms acquire electrons from Fe and Co atoms. The electron transfer direction between FeCoPt‐2 and FeCoPt‐4 has changed, demonstrating that the electronic structure of FeCoPt is effectively adjusted by Co content. Usually, the extra electrons for Pt atoms in FeCoPt‐4 might weaken its adsorption of intermediate products in methanol oxidation, thus promoting its electrocatalytic efficiency [32, 33]. And for FeCoPt‐2, the accumulated electrons in Fe atoms can enhance the magnetic field response, which is beneficial to raise its electrocatalytic performance by external magnetic field.

The electrocatalytic MOR performance for FeCoPt together with the magnetic field effect were explored by cyclic voltammogram (CV). The electrocatalytic activity of FeCoPt‐4 is 0.9 A/mg_Pt_, the highest among all the samples and 7.16 times larger than that of commercial Pt/C (Figure 2a). As shown in Figure S4a,b, the electrochemical active surface areas (ECSA) of the five FeCoPt samples and commercial Pt/C were measured and calculated from the hydrogen adsorption/desorption curves. The results show that FeCoPt‐4 exhibits the highest specific activity among the five FeCoPt samples. Moreover, its specific activity is 28 times that of Pt/C, demonstrating that the intrinsic activity of active sites for FeCoPt are superior to those of commercial Pt/C (Figure S4c). The analysis of electron transfer in FeCoPt‐4 explains its highest MOR activity, since the accumulated electrons facilitate CO desorption. The electrocatalytic durability test has also confirmed this hypothesis. As shown in Figure 2b, the decreases of peak current for FeCoPt‐4 is only 10.2% and 14.3% after ADT test without a magnetic field for 1000 and 2000 cycles, respectively. In contrast, the activities of FeCoPt‐2 and FeCoPt‐3 are reduced by 40.2% and 23.2% for 1000 cycles, respectively (Figure S5). Therefore, owing to the suitable Co content, FeCoPt‐4 possess the best activity and stability under the current acidic circumstance. Its MOR performance has exceeded other reported L1_0_‐phase Pt‐based alloys and Pt‐based alloys loaded on carbon substrates in the same experimental conditions (Figure 2c and Table S1) [34, 35, 36, 37, 38].

**FIGURE 2:**
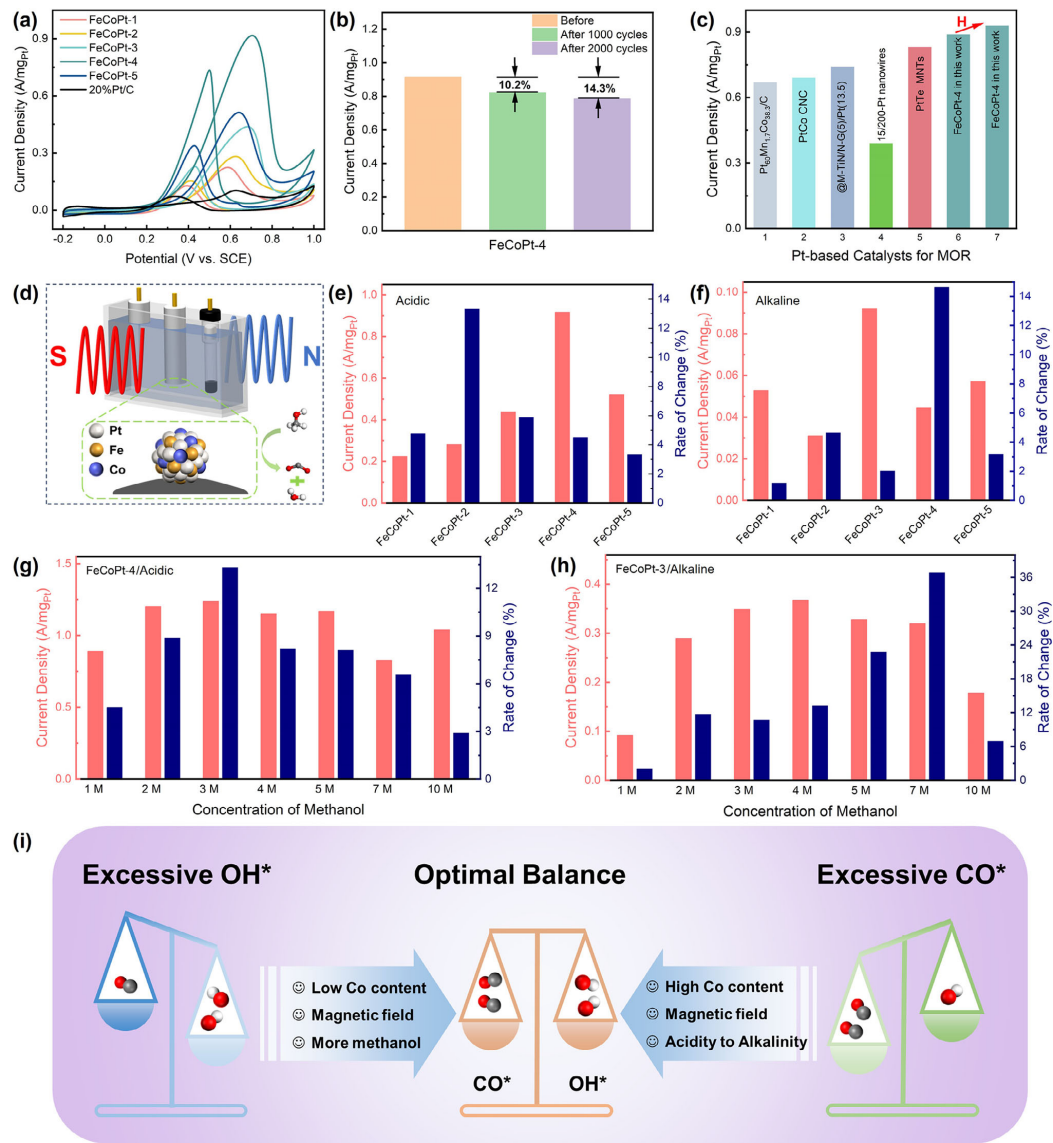
(a) CV curves in the acidic methanol solution and (b) the ADT experiments of 1000 and 2000 cycles for FeCoPt‐4 without magnetic field. (c) Comparison of the MOR activity. (d) Schematic diagram of the magnetic‐field‐assisted electrochemical testing system. The currents of I_f_ peak as well as the R(H) under 8000 Oe magnetic fields in (e) acidic methanol solution and (f) alkaline methanol solution. The relationship between methanol concentration and currents of I_f_ peak as well as the R(H) under 8000 Oe magnetic fields (g) for FeCoPt‐4 in 0.5 m H_2_SO_4_ and (h) for FeCoPt‐3 in 1.0 m KOH. (i) The balance model of MOR concerning methanol concentration, Co content, and acidity/alkalinity.

The structure stability has also been confirmed by characterizing the cycled samples with TEM and XRD. As shown in Figure S6, the morphologies of FeCoPt‐4 before and after ADT experiments are very similar. After ADT experiment, the edge and corner of FeCoPt nanoparticles are sharper and clearer, while the nanoparticles are distributed on the carbon substrate steadily. The distinct lattice fringes in HRTEM illustrate typical (110) and (001) crystal planes of L1_0_‐phase structure. Intensity profiles for the green boxes display the alternating atomic columns, which are attributed to Pt‐rich (bright) and Fe/Co‐rich (dim) atomic columns, respectively. EDS measurements of three regions for FeCoPt‐4 demonstrate that 3% Fe and 5% Co atoms are lost during ADT experiments (Table S2). The XRD patterns before and after ADT experiments demonstrate no obvious difference, further confirming the L1_0_‐phase structure stability (Figure S7).

The increasing Co content not only tune the electron transfer direction in FeCoPt samples, but also changes the spin‐dependent electronic structure and the magnetic properties. The M‐H curves of FeCoPt‐1, FeCoPt‐2, FeCoPt‐3 and FeCoPt‐4 have been collected before evaluating the magnetic field response in the electrochemical reaction. The hysteresis loops demonstrate the samples are all ferromagnetic at RT (Figure S8). The M_s_ and H_c_ for FeCoPt‐2 are the largest, while the values for FeCoPt‐4 are quite small compared to other samples (Table S3).

Lattice defects and structural disorder, related with grain size and lattice strain, are well‐known strategy to modulate the electronic structure and the final property. The variation of Co content simultaneously changes these parameters, making it necessary to clarify their effect on electrocatalytic activity and magnetic properties. The grain size and lattice strain are calculated by Scherrer formula and by Bragg diffraction formula according to the XRD data, respectively. First, Table S4 illustrates that the grain sizes of the five samples fluctuate with increasing Co content. The samples with the largest and smallest grain sizes are FeCoPt‐4 and FeCoPt‐5, while the mass activities of FeCoPt‐4 and FeCoPt‐5 are the highest and the second highest. Considering the magnetic properties shown in Figure S8, FeCoPt‐2 possesses the second largest grain size, but it shows the highest M_s_ and H_c_. Therefore, the effect of grain size on magnetic and catalytic properties of FeCoPt sample is not distinct. Second, compared to the (111) peak of L1_0_‐FePt in the standard PDF card (No. 45–1359), the (111) peaks of L1_0_‐FeCoPt samples shift to higher angles due to the formation of alloying phase between Pt, Fe and Co atoms. Since the size of Co atom is smaller than those of Fe and Pt atoms, the increase of Co content produces compressive strain and decreases the lattice constant. The lattice strain values of FeCoPt‐1, FeCoPt‐2, FeCoPt‐3, FeCoPt‐4, FeCoPt‐5 are −1.1%, −1.3%, −2.2%, −2.1%, −2.5%, respectively (Table S4). It is known that lattice strain influences the catalytic property, and the best performance usually appears with a moderate lattice strain [39]. The best catalysts in acidic and alkaline methanol solution are FeCoPt‐4 and FeCoPt‐3, which show that lattice strain might be related with the catalytic property. Considering that methanol concentration and solution pH can change the catalytic performance, the ability to produce OH* on Co sites is more pronounced in this work. On account for magnetic property, the lattice strain shows no direct relationship with M_s_ and H_c_. Therefore, both grain size and lattice strain have no direct effect on the magnetic properties. The grain size does not influence the catalytic property, while lattice strain might be related with the catalytic performance.

Then, the electrocatalytic MOR performance of FeCoPt samples is tuned by an 8000 Oe magnetic field generated with an electromagnet. Here, the magnetic field response can be expressed by the rate of change, R(H), which is defined as:

(1) $$R\;\left(H\right)=\frac{I\left(H\right)-I\left(0\right)}{I\left(0\right)}\;\times 100\%$$

where H is the magnetic field strength, I(0) and I(H) are the I_f_ peak currents in CV curves without and with magnetic fields. Figure 2d and Figure S9 show the schematic diagram and home‐made magnetic‐field–assisted measurement system employed in this work, respectively. The activity in acidic methanol solution for the five samples is acquired from the first oxidation peak (I_f_) in CV curves. As shown in Figure 2e, not only the activity but also the magnetic field response obeys a volcano shape with respect to the Co content, where the R(H) value of FeCoPt‐2 is 13.3%, the highest among all the samples. Replacing the acidic electrolyte with alkaline electrolyte can overcome some disadvantages that would reduce the electrocatalytic performance in acidic solution. One improvement is that the corrosion effect of the metal catalyst is alleviated in alkaline solution. Another is that the reaction kinetics is faster since the abundant and available OH^−^ ions in alkaline solution provide more radicals for the methanol oxidation. Therefore, the enhancement of MOR performance under a magnetic field has been further evaluated in alkaline methanol solution (Figure S10 and Figure 2f). Similar to the acidic circumstance, the electrocatalytic performance and the R(H) obey volcano shape plots with respect to the change of Co content in alkaline methanol solution. The best catalyst is FeCoPt‐3 with the highest mass activity, while FeCoPt‐4 possesses the best magnetic response as high as 14.7%. FeCoPt‐2 and FeCoPt‐4 are selected to study the effect of magnetic field strength on R(H) in acidic and alkaline media, respectively. As shown in Figure S11, the R(H) of both samples show a monotonic increasing trend with enhancing magnetic field strength. No matter in acidic and alkaline media, the magnetic field is beneficial to increasing the MOR performance.

Co content in FeCoPt samples strongly affects the activity and the magnetic field response. Meanwhile, the highest activity and the highest magnetic field response do not simultaneously appear in one sample, which is also observed in CoPt electrocatalytic system [3]. For example, FeCoPt‐4 shows the highest electrocatalytic activity in acidic solution, while FeCoPt‐3 with less Co content is the best in alkaline solution. On the other hand, FeCoPt‐2 possess the largest magnetic field response in acidic solution, but FeCoPt‐4 with more Co content exhibits the largest in alkaline solution. The underlying mechanism, governed by multi factors, is fascinating. It is known that the half‐reaction that occur on the electrodes are different in acidic and alkaline solutions (Formulas 2–6).

Acidic:

(2) $$CH_{3}\mathrm{OH}+H_{2}O\to CO_{2}+6H^{+}+6e^{-}$$

(3) $$3/2O_{2}+6H^{+}+6e^{-}\to 3H_{2}O$$

Alkaline:

(4) $$CH_{3}\mathrm{OH}+6OH^{+}\to CO_{2}+5H_{2}O+6e^{-}$$

(5) $$3/2O_{2}+3H_{2}O+6e^{-}\to 6OH^{-}$$

The FeCoPt catalysts follow bifunctional mechanism for MOR process, where the Pt sites facilitate the dehydrogenation of CH_3_OH into CO* and the M (M = Fe, Co) sites are beneficial to splitting H_2_O into OH*. The rate‐determining step is oxidizing CO* with OH* intermediates into CO_2_. Such synergistic effect can be expressed in the following Formula (6).

(6) $$\mathrm{Pt}-\mathrm{CO}+\mathrm{HO}-M\to Pt^{*}+M^{*}+CO_{2}+H^{+}+e^{-}$$

When the reaction of CO* and OH* reaches balance, the I_f_ peak in the CV curve is acquired at a specific potential. According to Le Chatelier's principle, if one of the conditions of the equilibrium system is changed, the equilibrium will shift in the direction opposite to this change. Considering the case without a magnetic field, the OH^−^ ions in alkaline methanol solution are much more than those in acidic methanol solution. The facile availability of OH^−^ ions makes it easier to transform OH^−^ ions to OH* intermediates in alkaline methanol solution; the demand for OH* is reduced to reach a new balance. Therefore, the highest electrocatalytic activity appears in FeCoPt‐3 sample with less Co content in the alkaline environment, while FeCoPt‐4 sample with more Co content shows the highest activity in the acidic environment.

The external magnetic field can break the equilibrium between CO* and OH*, which is reflected by the magnetic field response. The magnetic field is proved to enhance OH* production and weaken the Pt─CO bond through the shift of I_f_ peak in the CV curve [3]. In situ Raman spectroscopy has proved that the magnetic field effectively promotes the formation of OH* species during MOR. Meanwhile, CO stripping curves confirm that the magnetic field plays a positive role in both CO adsorption and reaction processes [28]. In acidic methanol solution, a volcano plot is observed, and the largest R(H) appears in FeCoPt‐2 instead of FeCoPt‐4. The FeCoPt‐4 sample already produces enough OH* for methanol oxidation owing to the 43% Co content. The external magnetic field only produces excessive OH* to occupy the active sites and hinder the CO* adsorption, leading to a weak magnetic field response [28]. But the Co content in FeCoPt‐2 is only 25%. There is much more requirement for OH* intermediates to react with CO*. Thus, applying a magnetic field to FeCoPt‐2 can enhance the electrocatalytic performance the most.

The trend of magnetic field response in alkaline methanol solution is opposite to that in acidic environment. In the acidic solution, OH* is produced from the electrolysis of water, whereas in the alkaline solution, OH^−^ is readily available in the solution [40]. The activity of FeCoPt‐3 is the highest, while the magnetic field response of FeCoPt‐4 is the largest despite the Co content is as high as 43%. This change is because the production of OH* is not difficult in alkaline environment, and the adjustment of Pt─CO bond under a magnetic field turns to be key to obtain the equilibrium. Therefore, the FeCoPt‐4 exhibits the largest magnetic field response. The analysis of the magnetic field effect in acidic and alkaline methanol solution explains why the largest magnetic field enhancement does not appear in the sample with the highest electrocatalytic activity.

The OH* production and Pt─CO bond strength are judged from the shift of the I_f_ peak in CV curves. As shown in Figure 2a and Figure S12, with increasing Co content, the I_f_ peak first shifts positively (to higher potentials) and then negatively (to lower potentials). Because Co sites can dehydrogenate water into OH*, the positive shift indicates enhanced OH* production and a weakened Pt─CO bond, which optimize the MOR performance of FeCoPt‐4. With more Co sites in FeCoPt‐5, OH* is in excess, leading to the negative shift of I_f_ peak. Therefore, FeCoPt‑4 exhibits the highest catalytic activity. It should be noted that the applied magnetic field enhances the activity of FeCoPt‐5 even though OH* is in excess in this sample. This proves that the magnetic field can not only tune OH* production but also modulate Pt─CO bond strength, resulting in enhanced catalytic activity.

The methanol concentration in the solution is also helpful to raise the reaction equilibrium and unveil the reaction mechanism under a magnetic field. Considering FeCoPt‐4 and FeCoPt‐3 respectively exhibit the maximum current density in acidic and alkaline circumstance, the methanol concentrations are adjusted from 1 to 10 m for these two samples. Both the activity and the magnetic field response show volcano shape plot with the variation of methanol concentrations. As shown in Figure 2g and Figure S13, when the CH_3_OH increases to 3 m, FeCoPt‐4 exhibit the best activity in an acidic environment. Meanwhile, the largest magnetic field response appears when the CH_3_OH is 3 m. As for FeCoPt‐3, the best performance and the largest magnetic field response in alkaline environment appear when the CH_3_OH are 4 and 7 m, respectively. Recently, we have conducted molecular dynamics for MOR simulation, demonstrating that the amounts of CH_3_OH and H_2_O molecules near the FePt catalyst surface are the same when the methanol is 4 m [41]. This molecular configuration approaches the optimal balance for MOR, resulting in the highest activity.

The mechanism explanation of methanol concentration can be drawn upon the effect of Co content based on Le Chatelier's principle. Considering in the acidic methanol solution, the diffusion process limits methanol supply at low concentrations (1 m), where abundant OH* adsorbates dominate the surface and block active sites. As the methanol concentration increases to 3 m, more methanol molecules reach the catalyst surface and provide more CO* intermediates to react with OH*, which drives MOR activity to an optimal equilibrium state [42]. With more methanol ranging from 3 to 10 m participated in the MOR process, the OH* intermediates become lacking, leading to a decrease of activity. The largest R(H) appears as 13.3% with 3 m methanol. By replacing acidic methanol solution with alkaline methanol solution, the highest activity is acquired with a larger methanol concentration (4 m). The R(H) value is drastically increased to 36.8% when the methanol concentration is 7 m, which is 31 times that of the solution with 1 m CH_3_OH (Figure 2h and Figure S14). The plentiful OH^−^ is directly provided from the alkaline solution, and methanol is the relatively minor reactant. The influence of methanol concentration is quite distinct to the activity, as more CO* adsorbates are produced for reaction. Moreover, the weakening of Pt─CO bond is more pronounced by external magnetic field due to the enough supply of OH*. Therefore, the methanol concentration in the alkaline solution for the highest activity and the largest magnetic field response is higher than in the acidic solution.

The mechanism for adjusting MOR activity is schematically illustrated in Figure 2i. The synergistic effect of both CO* and OH* intermediates decides the optimum balance [42]. Methanol concentration and acidity/alkalinity are the external factors for the MOR process. The Co content in FeCoPt decides the intrinsic electronic structure. The magnetic field is able to tune the spin‐dependent electronic structure, further affecting the adsorption and reaction of functional groups in MOR process. To completely understand the regulation of Co content on the spin‐dependent electronic structure, especially the d‐band center and the net spins, first‐principles calculations on L1_0_‐FeCoPt have been carried out. The optimized atomic structures of L1_0_‐FeCoPt are enumerated in Figure S15.

The projected density of states (PDOS) of the Fe, Co and Pt d‐orbitals for the L1_0_‐FeCoPt models are calculated without considering spin state, respectively (Figure 3a–c). The increasing Co will occupy both Fe sites and Pt sites, alter the coordination environment of the surround atoms, and finally change the electronic structure. With increasing Co content, the d‐band center of Fe element increases firstly and then reduce. The highest d‐band center lies at −0.498 eV in FeCoPt‐2. This suggests that more empty d‐orbitals are available for hybridization, which enhances the bonding between metal and OH* adsorbate [43]. On the contrary, the d‐band center of Co decreases and then increases with the variation of Co content. The d‐band center in FeCoPt‐2 is the lowest, indicating the bonding ability at Co sites is weakened. In the case of Pt, the differences between d‐band centers and Fermi level are more than −2.0 eV. With increasing Co content, the d‐band center of Pt shifts toward higher energy. The d‐band center of Pt in FeCoPt‐2 is positioned at a lower middle position, while those in FeCoPt‐3 and FeCoPt‐4 are higher. Previous XPS results have shown that the electrons belonging to Pt and Co atoms are inclined to transfer to Fe atoms for FeCoPt‐2 sample. This is consistent with the negative shift of the d‐band center in FeCoPt‐3, since electrons have moved to Fe atoms. Moreover, the electron transfer direction in FeCoPt‐4 has changed. The calculated electronic structure for Pt single crystal demonstrates that the d‐band center is ‐2.35 eV, which is between FeCoPt‐3 and FeCoPt‐4 (Figure S16). The change of d‐band center by varying Co content is the driving force to tune the electron transfer direction in magnetic Pt‐based alloys. Usually, a Pt‐5d orbital with high energy is detrimental to the desorption of carbon intermediate, but a Pt‐5d orbital with too low energy would inhibit the adsorption of initial methanol molecules [32, 33]. Therefore, a moderate d‐band center is required to optimize the coverage of CO* intermediate to react with OH*, thus promoting the MOR performance in FeCoPt alloys [44, 45].

**FIGURE 3:**
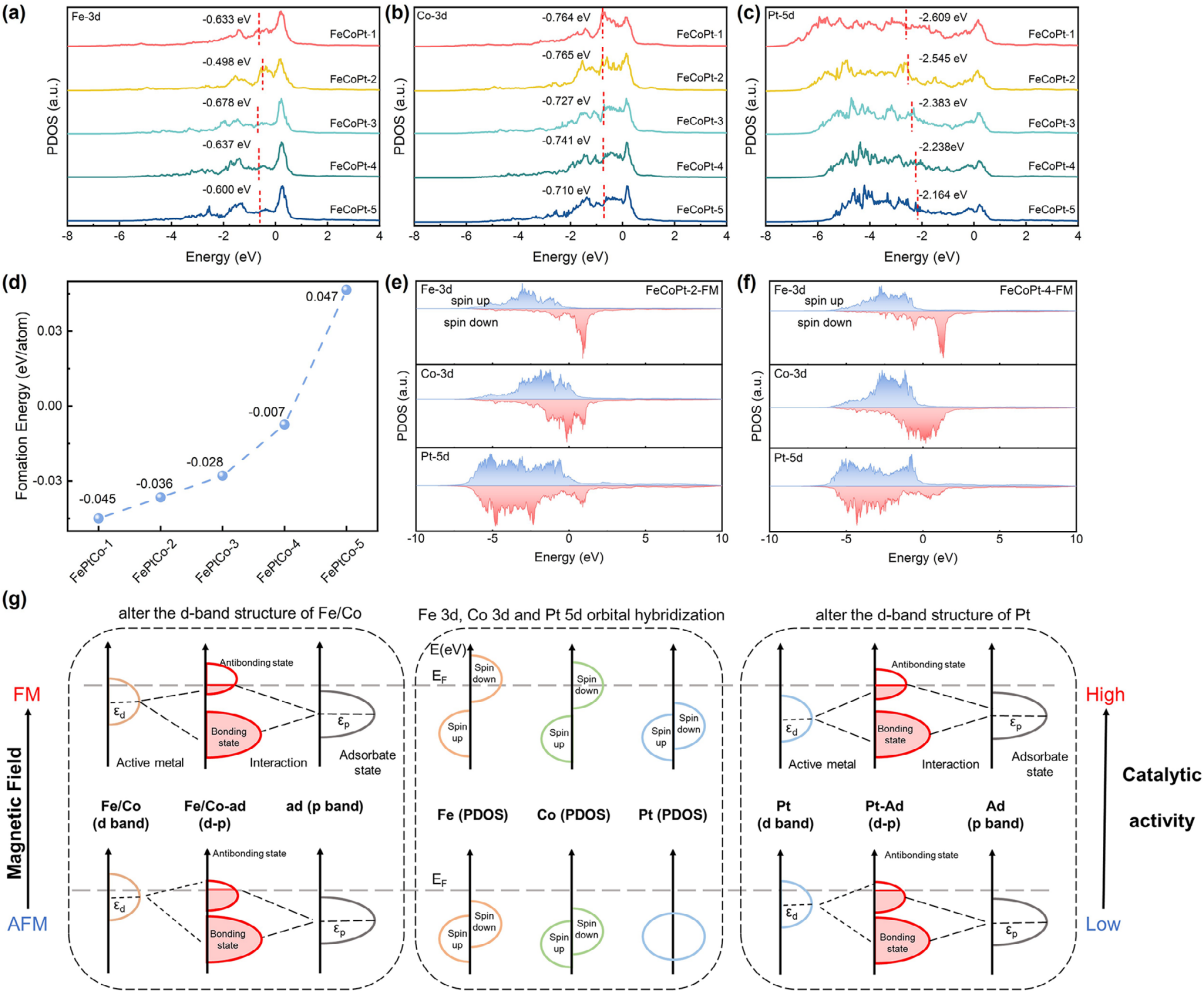
The PDOS of the (a) Fe 3d‐band, (b) Co 3d‐band, (c) Pt 5d‐band in the normal L1_0_‐FeCoPt, respectively. (d) Formation energies of L1_0_‐FeCoPt. The PDOS of the Fe 3d‐band, Co 3d‐band, Pt 5d‐band in the spin‐polarized (e) FeCoPt‐2, (f) FeCoPt‐4, respectively. (g) Mechanism for the movement of the d‐bands for Fe, Co and Pt.

The formation energy is calculated to check the rationality of these L1_0_‐phase structures (Figure 3d). The FeCoPt‐1 model is the most stable structure since its formation energy is −0.045 eV/atom, lower than the other four models. Increasing Co content would raise the formation energy. The formation energy of FeCoPt‐5 model becomes positive, indicating it is not a stable structure. In order to form L1_0_ phase structure, the Pt content should be kept between 45% and 55% [46]. The FeCoPt‐5 model is far away from this range, leading to such an unstable structure and abnormal d‐band centers compared to other models.

To gain deeper insight for the magnetic field response, the evolution of the spin‐dependent electronic structure in L1_0_‐FeCoPt is calculated by considering the spin polarization in the projected density of states (PDOS) for FeCoPt‐2 and FeCoPt‐4 (Figure 3e,f). The symmetries of PDOS in spin‐up and spin‐down states are distinctly broken for Fe and Co elements. Interestingly, the spin‐up and spin‐down states for Pt are slightly different, indicating the existence of small spins.

The net spins of each element in the single unit are calculated based on the PDOS structures with spin‐up and spin‐down configurations and shown in Table S5. The results indicate that FeCoPt‐2 possesses the highest effective magnetic moment for Fe elements. The greater the magnetic moment, the stronger the magnetic field response [28]. The Fe site in FeCoPt‐2 exhibits the strongest magnetic field response. Meanwhile, the Co net spins first increase and then decrease with increasing Co content. The highest Co net spins appear in FeCoPt‐4 with a smaller Fe net spin. The sum of Fe and Co net spins is 0.34, 0.46, 0.51, 0.58, 0.43 µ_B_. The drop of net spins in FeCoPt‐5 may be the result of its unstable structure. Therefore, varying Co content can continuously change the spin states as well as the magnetic field response for MOR process. The net spins of Pt are oppositely aligned to Fe/Co spins, with the value being less than 1/5 of the sum for Fe and Co spins. The calculated values are consistent with previous experimental and theoretical findings [20]. The highest magnetic field responses are FeCoPt‐2 in acidic environment and FeCoPt‐4 in alkaline environment. The behind mechanism is the combination of the change of spins in L1_0_‐FeCoPt samples and the solution environment.

The electronic structure is highly related with the model structure. The effect of disorder effect on the d‐band center and net spin has been studied on FeCoPt‐2 model. As illustrated in Figure S17, three approaches are considered to simulate the disorder effect: (1) Atomic exchange. (2) Atomic displacement. (3) Point defect. The formation energy analysis demonstrates that the original model is the most stable structure. Atomic exchange and atomic displacement will decrease the structure stability, while the point defect of Pt atom will make the structure unstable. Tables S6 and S7 exhibit the calculated d‐band centers and net spins. The atom exchange will move the d‐band centers of Fe and Co away from the Fermi level, while shift that of Pt toward the Fermi level. These shifts suggest a weakened OH* adsorption and an enhanced CO* adsorption on the surface, which are detrimental to the catalytic property. The sum of net spins for Fe and Co increases, which may reflect an enlarged magnetic field response. As for the atom displacement, the d‐band centers of Fe, Co and Pt simultaneously shift away from the Fermi level, while net spins are decreased. These also lead to lower catalytic property. In the case of point defect of Pt atom, the d‐band centers and net spins change a lot due to its unstable structure.

With tunable net spins in five FeCoPt samples, the splitting of spin‐up and spin‐down electronic band structure is the key to generate magnetic field response. With the appliance of magnetic field, the electronic structure is spin‐polarized (Figure 3e,f). The d‐band centers for spin‐up and spin‐down states are demonstrated in Table S8. The splitting distance of d‐band centers for Fe element is as large as 2.889 eV in FeCoPt‐2, while the distances are smaller in other models (Table S9). For both Co and Pt elements, the splitting distance of d‐band centers enlarges with more Co contents, which increases the difference of spin‐up and spin‐down electrons. Since the spin‐down electrons are closer to the Fermi level, they act as the “hot electrons” that act as the main role in participating in catalysis. The spin‐down electrons positively shift with increasing Co content, providing more vacant orbitals to couple with intermediates such as CO* and OH* [44]. Therefore, the variation of spin state by magnetic field provides a facile approach to tune the adsorption of intermediates during the MOR process.

To build a relationship between magnetic field strength and catalytic property by theoretical calculation is an important issue. Normalizing the magnetic moment by the saturation moment in the initial magnetization curve, the experimental values are corresponded to the theoretical degree of spin polarization (Table S10). Thus, the electronic structures of five typical magnetic field can be simulated by setting the magnetic moment value. With the magnetic field increasing from 3500 to 4500 Oe, the d‐band center of Pt‐spin down band decreases gradually (Figure S18). Then the value rises and stabilizes with larger magnetic field. The decrease of d‐band center can weaken the adsorption of CO* on Pt sites, which is beneficial to accelerating MOR process. The trends for Fe‐ and Co‐spin down band are inverse, which can optimize the OH* adsorbate. Therefore, the theoretical analysis demonstrates that magnetic field strength has a positive correlation with catalytic performance.

To investigate the effect of spin on the adsorption of the reaction intermediate *OH, the adsorption energies for OH* in FeCoPt‐4 without and with spin are exhibited in Figure S19. It is obviously found that the adsorption at Fe site is strengthened, while the adsorption at Co site is weakened. Considering that Co content is more than Fe in L1_0_‐FeCoPt, the adsorbed OH* intermediates are easily removed from the active sites, which enhance the production of OH* radicals. Meanwhile, the adsorption energy at Pt sites at ferromagnetic state becomes negative compared to normal state. This change also facilitates the production of OH*, promoting the total MOR process. Spin‐induced orbital interactions typically result in changes in energy levels. The band adjustment mechanism under magnetic field is illustrated in Figure 3g. The increased magnetic moments of the 3d orbitals of Fe and Co not only induce spin splitting in the energy bands of Fe and Co, but also trigger spin splitting in the Pt bands via exchange coupling. For Fe, Co and Pt, the spin‐down bands shift toward the Fermi level (E_F_), while the spin‐up bands shift in the opposite direction [47]. This spin splitting concurrently modifies the d‐band structures of all three elements, with the d‐band changes in Fe and Co being more significant than those in Pt. Consequently, although the modification of Pt d‐band enhances *CO adsorption, the Fe and Co dominated enhancement of *OH adsorption is more pronounced, thereby improving the overall catalytic activity.

The favorable adsorption configurations of CH_3_OH and their sequential intermediates on the slabs are presented in Figure 4a. The ferromagnetic (FM) and antiferromagnetic (AFM) configurations reflect two states with and without net spins, which mimic the conditions before and after applying external magnetic fields. The reaction of CO* and OH* is the rate‐determining step. The Gibbs free energy profiles manifest the energy barrier at the rate‐determining step for FeCoPt‐4‐FM is only 1.22 eV, lower than that of normal FeCoPt‐4 (1.83 eV). The optimization of the reaction barrier indicates that methanol oxidation takes place more easily on ferromagnetic state. The calculation firmly verifies that net spins resulted from external magnetic fields effectively facilitate the process of MOR by lowering the activation barrier.

**FIGURE 4:**
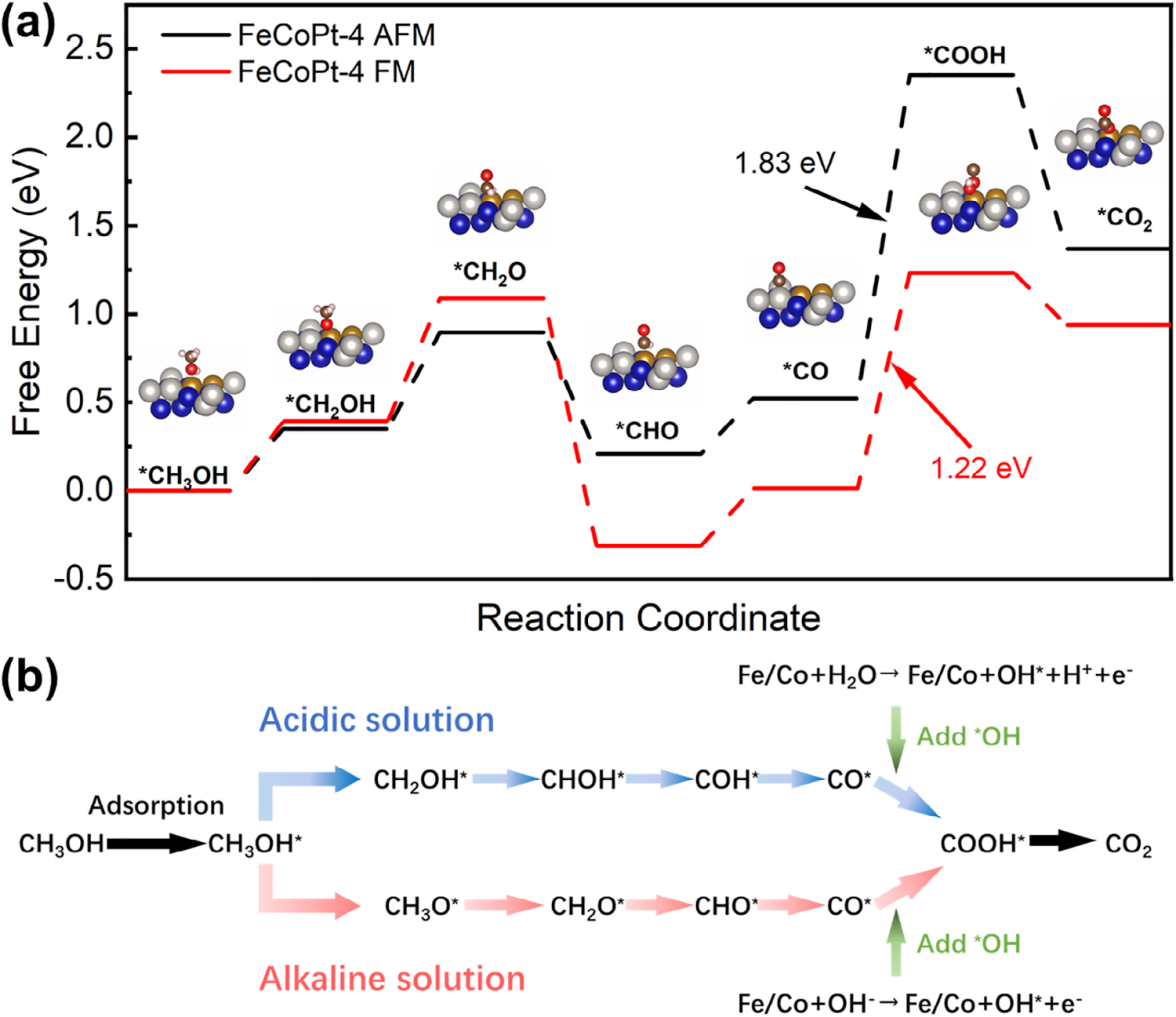
(a) Multistep Gibbs free energy of MOR for FeCoPt‐4 with AFM and FM states at zero potential. (b) The MOR mechanism.

The reaction pathways of the MOR process differ between acidic and alkaline environments, primarily due to the varying origin of OH* intermediate, which leads to differences in the reaction mechanisms under magnetic fields (Figure 4b). In acidic environment, protons are abundant, and OH* can only be acquired from H_2_O, surface is prone to poisoning by intermediates like CO. In alkaline environment, the rich OH^−^ ions can provide enough OH* to remove the CO* intermediate. The magnetic field is able to tune the electronic structure in L1_0_‐FeCoPt, thus, the equilibrium between CO* and OH* varies, and a higher reaction performance appears. But the acidity and alkalinity are also important and non‐ignorable factors that change the adsorption behavior of the intermediates. It can be concluded that the magnetic field effect is not only related with the spin catalysts, but also the reaction environment, especially the adsorbed intermediates.

## 3 Conclusion

In summary, FeCoPt samples have been successfully synthesized via the hydrothermal method and the annealing process at 700°C. By controlling the Co content, the electronic band structure and magnetic properties of the FeCoPt alloys can be continuously regulated. FeCoPt‐2 exhibited the highest saturation magnetization and coercivity among all samples, resulting in the largest magnetic field response in acidic methanol solution. On the other hand, FeCoPt‐4 exhibits the highest activity, which is 7.16 times of commercial Pt/C. According to the Sabatier Principle, the amounts of CO* and OH* intermediates decide the reaction equilibrium, which are related with the methanol concentration, acidity and alkalinity of the solution. Meanwhile, external magnetic fields also adjust the reaction equilibrium by tuning the OH* amount and Pt‐CO bond strength. In a mixed solution of 0.5 m H_2_SO_4_ and 3 m CH_3_OH, it exhibits the best MOR activity and strongest magnetic field response. In a mixed solution of 1 m KOH and 7 m CH_3_OH, the FeCoPt‐3 catalyst demonstrates the highest rate of change (36.8%). First‐principles calculations reveal that increasing Co content shifts the Pt‐5d orbital d‐band center toward the Fermi level. Magnetic field‐induced spin splitting of Fe and Co 3d orbitals causes the downspin band to shift toward the Fermi level. The upward shift of Fe, Co and Pt d‐band centers simultaneously optimize the binding strength for the intermediates. The concept that optimal MOR performance is achieved when the concentrations of CO* and OH* reach equilibrium in the reaction system offers a novel perspective for exploring the competitive interactions between functional groups in magnetically controlled organic molecular reactions. The operando experiments to quantify the CO* and OH* may be future work to develop a standard technique to deepen the understanding of intermediate process. Generally, this study elucidates how the synergistic interaction between d‐band center shifts and dual‐function mechanisms under magnetic fields enhances the MOR process, providing guidance for designing spin catalysts for DMFCs.

## Author Contributions

**Qiqi Mo**: conceptualization, methodology, software, formal analysis, visualization, writing – original draft. **Kaijie Liu**: conceptualization, software, data curation. **Ruixi Liu**: investigation, validation. **Yong Juan**: conceptualization, methodology, investigation. **Sihan Wang**: conceptualization, investigation. **Jialong Liu**: investigation, funding acquisition, writing – original draft, project administration. **Wei Wang**: data curation, supervision, validation, project administration, funding acquisition, writing – review and editing, methodology.

## Conflicts of Interest

The authors declare no conflicts of interest.

## Supporting information




**Supporting File**: advs78112‐sup‐0001‐SuppMat.docx.

## Data Availability

The data that support the findings of this study are available on request from the corresponding author upon reasonable request.
