# Supplementary material for: Magnetic‐Field‐Tuned Spin‐Dependent D‐Band and Bifunctionality in L10‐FeCoPt for Enhanced Methanol Oxidation
Source: Adv Sci (Weinh). 2026 Sep 30:e78112. Online ahead of print. doi: 10.1002/advs.78112 (PMC13627075; doi:10.1002/advs.78112)
Supplement: Supplementary file 1 [file ADVS-9999-e78112-s001.docx]

**Supporting Information**

**Magnetic-Field-Tuned Spin-Dependent d-Band and Bifunctionality in L1₀-FeCoPt for Enhanced Methanol Oxidation**

Qiqi Mo, Kaijie Liu, Ruixi Liu, Yong Juan, Sihan Wang, Jialong Liu^*^, Wei Wang^[1]*^

Department of Physics and Electronics, School of Mathematics and Physics, Beijing University of Chemical Technology, Beijing 100029, China

**1. Experimental Section**

**1.1 Chemicals**

Iron (III) acetylacetonate [Fe(acac)_3_], platinum (II) acetylacetonate [Pt(acac)_2_], cobalt (II) acetylacetonate [Co(acac)_2_], N, N-dimethylformamide (DMF) were purchased from Macklin Company. Cabot carbon (XC-72) was purchased from Suzhou Sinero Technology Co., Ltd. Commercial Pt/C (20% Pt) catalysts were purchased from Innochem Company. Polyvinyl pyrrolidone (PVP) was purchased from Shandong Xiya Chemistry Industry Ltd. The reagents were of analytic grade and used without modification.

**1.2 Synthesis of FeCoPt samples**

The FeCoPt samples were prepared by hydrothermal and annealing method with different element ratios of Fe, Co and Pt. In a typical synthesis procedure of FeCoPt-4 sample, 4.2 mg of Fe(acac)_3_, 12.2 mg of Co(acac)_2_, 14 mg of Pt(acac)_2_ and 70 mg of PVP were dissolved in 20 ml of DMF. The solution was sonicated for more than 30 min to mix the precursors thoroughly. Then, the solution was transferred to a 25 ml Teflon-lined stainless-steel autoclave and reacted at 180 ºC for 12 h before cooling to room temperature (RT). The powder sample was obtained by washing with deionized water and ethanol repeatedly together with drying. Then, 7 mg of the powder sample was mixed with 5 mg Cabot carbon (XC-72), followed by annealing in a quartz boat at 700 °C in N_2_ ambient for 6 h. Using the similar method but varying the mass of the Co(acac)_2_ to 3.1, 6.1, 9.2 and 15.3 mg, FeCoPt-1, FeCoPt-2, FeCoPt-3 and FeCoPt-5 were synthesized, respectively.

**1.3 Characterization**

The crystal structure, morphology, and elemental distribution of FeCoPt was analyzed by using X-ray diffraction (XRD, Malvern Panalytical Aeris) and transmission electron microscopy (TEM, JEOL 2200FS) equipped with an energy-dispersive X-ray spectroscope (EDS, Oxford). The aberration corrected high angle annular dark field scanning transmission electron microscopy (HAADF-STEM) was performed on Thermo Fisher Spectra 300. The elemental composition and valence were analyzed by X-ray photoelectron spectroscopy (XPS, VG ESCALAB 220 IXL). The hysteresis loop was measured via a physical property measurement system (PPMS, Quantum Design).

**1.4 Electrochemical Measurements**

The electrochemical measurement was carried out on a CH Instruments 600E electrochemical workstation with a three-electrode system at RT. The counter electrode and reference electrode were a platinum wire and saturated calomel electrode (SCE), respectively. To prepare the working electrode, a glassy carbon electrode was firstly polished and washed with deionized water. Next, 5 mg of samples were ultrasonicated and dissolved in 5 ml mixed solution composed of deionized water and anhydrous ethanol. Subsequently, 30 μl of the mixed solution and 5 μl of 5% Nafion solution were sequentially dropped onto the glassy carbon electrode and dried in air.

The methanol oxidation reaction was carried out by cyclic voltammetry (CV) with the potential scans between -1 and 0.2 V (vs. SCE electrode) at a rate of 50 mV/s. The alkaline methanol solution was composed of 1 M KOH and 1 M CH_3_OH while the acidic methanol oxidation was composed of 0.5 M H_2_SO_4_ and 1 M CH_3_OH. The stability experiment was conducted by accelerated durability test (ADT) in the methanol oxidation at RT for 1000 cycles. A homemade multi-field electrocatalytic system with variable magnetic fields was applied to explore their influences on MOR performance. After the CV curves reached stable, the 8000 Oe magnetic fields were applied to measure the electrocatalytic properties.

**1.5 First-Principles Calculation**

The theoretical calculations were carried out via Density Functional Theory (DFT) using the Generalized Gradient Approximation (GGA) and Perdew-Burke-Ernzerh of (PBE) functions in the VASP^[1]^. The FeCoPt bulk structure and 2 × 2 FeCoPt (111) slab with the L1_0_-phase structure were constructed to study the electronic structure and simulate the MOR multistep process under magnetic fields. Different adsorption configurations of CH_3_OH and other intermediates were considered and calculated to obtain the most equilibrium sites. A vacuum slab was set as 20 Å to avoid the vertical boundary effect^[2]^. To represent the bulk properties of FeCoPt and FeCoPt alloy, the bottom layer was constrained and the remaining top layers together with the adsorbates were relaxed^[3]^. The cut-off energy was set to 500 eV and a 3 × 3 × 1 Monkhorst-Pack k-point mesh was sampled. Structure optimization was conducted until the total energy convergence reached 10^−5^ eV. The spin polarization was also taken into consideration. To investigate the effect of magnetic field on MOR, the antiferromagnetic (AFM) and ferromagnetic (FM) states were respectively considered to imitate the situation without and with magnetic field during each reaction step. The following formula was used to calculate adsorption energies of CH_3_OH and intermediates on the slabs,

ΔG=G_ad + substrate_ – G_substrate_ – G_ad_	(1)

Here, the G_ad + substrate_ and G_substrate_ implied the Gibbs free energy of the system with and without the adsorbate, while G_ad_ represented the Gibbs free energy of the adsorbate. Specifically, the energy of a single H atom (G_H_) is acquired by halving the energy of a hydrogen molecule ($E_{H_{2}}$). When evaluating the Gibbs free energy in the multistep MOR process, the zero-point energy and vibrational entropy part were considered, which is defined as:

G = E + E_zpe_ – TΔS	(2)

where E_zpe_ and TΔS represent the zero-point energies and vibrational entropy energies of the molecules at the adsorption states, respectively. T is set to the RT of 298 K^[4]^.

References

[1]	G. Kresse, J. Furthmüller. Efficient iterative schemes for ab initio total-energy calculations using a plane-wave basis set. *Phys. Re. B* **54** (1996) 11169.

[2]	H. Huang, H. H. Wu, C. Chi, B. L. Huang, T. Y. Zhang. Ab initio investigations of orthogonal ScC_2_ and ScN_2_ monolayers as promising anode materials for sodium-ion batteries. *J. Mater. Chem. A* **7** (2019) 8897.

[3]	J. Li, H. Huang, X. X. Cao et al. Polyvinylpyrrolidone gel based Pt/Ni (OH)_2_ heterostructures with redistributing charges for enhanced alkaline hydrogen evolution reaction. *Chem. Eng. J.* **416** (2021) 127677.

[4]	V. Wang, N. Xu, J. C. Liu, G. Tang, W. T. Geng. High-throughput computational screening of two-dimensional semiconductors. *Comput. Phys. Commun.* **267** (2021) 108033.

**2. Figures and Tables**

**Figure S1.** (a) Low-resolution TEM image for pristine FeCoPt sample. (b) SAED pattern and (c)-(d) High-resolution TEM images for FeCoPt.

**Figure S2.** Low-resolution TEM image and high-resolution TEM image for (a_1_-a_3_) FeCoPt-1, (b_1_-b_3_) FeCoPt-2, (c_1_-c_3_) FeCoPt-3, (d_1_-d_3_) FeCoPt-4, and (e_1_-e_3_) FeCoPt-5, respectively. Inset is SAED pattern.

**Figure S3.** The (111) peak shift for the five annealed FeCoPt samples.

**Figure S4.** (a) CV curves collected in 0.5 M H_2_SO_4_. (b) The ECSA and (c) the specific activities of the five FeCoPt samples and commercial Pt/C.

**Figure S5.** The stability test of FeCoPt-2 and FeCoPt-3 by ADT experiments for 1000 cycles without magnetic field.

**Figure S6.** The TEM images for FeCoPt-4 (a-c) before and (e-g) after ADT experiments for 2000 cycles. (d, g) The intensity line profile for the green boxes in (c) and (g), respectively.

**Figure S7.** XRD patterns of FeCoPt-4 before and after ADT experiments for 2000 cycles.

**Figure S8.** (a, b) M-H curves and (c) corresponding M_s_ as well as H_c_ of FeCoPt-1, FeCoPt-2, FeCoPt-3 and FeCoPt-4 at 300 K.

**Figure S9.** The home-made magnetic-field-assisted measurement system.

**Figure S10.** CV curves of FeCoPt samples in alkaline methanol solution.

**Figure S11.** The rate of change for I_f_ peaks vs. magnetic fields for (a) FeCoPt-2 in 0.5 M H_2_SO_4_ and (b) FeCoPt-4 in 1 M KOH.

**Figure S12.** The peak potential shifts of the first oxidation peaks for the five FeCoPt samples compared with FeCoPt-1.

**Figure S13.** CV curves of FeCoPt-4 in 0.5 M H_2_SO_4_ with different concentrations of methanol.

**Figure S14.** CV curves of FeCoPt-3 in 1 M KOH with different concentrations of methanol.

**Figure S15.** Atomic structures of FeCoPt-1, FeCoPt-2, FeCoPt-3, FeCoPt-4 and FeCoPt-5, respectively.

**Figure S16.** The PDOS of the Pt 5d-band in the FCC Pt.

**Figure S17.** (a) Atomic model of FeCoPt-2 illustrating three disorder effect and (b) corresponding formation energy.

**Figure S18**. The d-band centers under different magnetic fields of (a) Fe, (b) Co, (c) Pt spin down band in FeCoPt-4.

**Figure S19.** The adsorption energy for OH* in FeCoPt-4 without and with spin.

**Table S1.** The experimental parameters for MOR and the catalytic performances.

| **Materials** | **Solution** | **Scanning rate**  **(mV/s)** | **Mass activity (A/mg_Pt_)** | **vs. Pt/C (Times)** | **Ref.** |
| --- | --- | --- | --- | --- | --- |
| Pt_60_Mn_1.7_Co_38.3_/C | 0.5M H_2_SO_4_+  1 M CH_3_OH | 50 | 0.67 | 1.90 | 34 |
| PtCo CNC | 0.5M H_2_SO_4_+  1 M CH_3_OH | 50 | 0.69 | 4.16 | 35 |
| @M-TiN/N-G(5)/Pt(13.5) | 0.5M H_2_SO_4_+  1 M CH_3_OH | 50 | 0.74 | 2.32 | 36 |
| 15/200-Pt nanowires | 0.5M H_2_SO_4_+  1 M CH_3_OH | 50 | 0.39 | 3.94 | 37 |
| PtTe MNTs | 0.5M H_2_SO_4_+  1 M CH_3_OH | 50 | 0.83 | 3.77 | 38 |
| FeCoPt-4 | 0.5M H_2_SO_4_+  1 M CH_3_OH | 50 | 0.90 | 7.16 | This work |

**Table S2.** The EDS results of FeCoPt-4 after ADT experiments.

| **Element** | **Region-1 (%)** | **Region-2 (%)** | **Region-3 (%)** | **Average (%)** |
| --- | --- | --- | --- | --- |
| Fe | 14.51 | 15.32 | 14.81 | 14.88 |
| Co | 37.34 | 37.34 | 37.79 | 37.49 |
| Pt | 48.16 | 47.33 | 47.40 | 47.63 |

**Table S3.** M_s_ and H_c_ of different samples.

| Sample | FeCoPt-1 | FeCoPt-2 | FeCoPt-3 | FeCoPt-4 |
| --- | --- | --- | --- | --- |
| M_s_/emug^-1^ | 28.6 | 32.1 | 21.7 | 22.5 |
| H_c_/Oe | 6700 | 14700 | 9300 | 4000 |

**Table S4.** Grain size and lattice strain for different samples.

| **Sample** | **FeCoPt-1** | **FeCoPt-2** | **FeCoPt-3** | **FeCoPt-4** | **FeCoPt-5** |
| --- | --- | --- | --- | --- | --- |
| Grain size/nm | 23.5 | 24.8 | 22.6 | 27.8 | 19.9 |
| Lattice strain | -1.1% | -1.3% | -2.2% | -2.1% | -2.5% |

**Table S5.** The densities of spin-up and spin-down states as well as the net spins of different samples.

| **Sample** | **Fe** | **Spin value (μ_B_)** | **Co** | **Spin value (μ_B_)** | **Pt** | **Spin value (μ_B_)** |
| --- | --- | --- | --- | --- | --- | --- |
|  | Up | 14.715 | Up | 14.645 | Up | 47.910 |
| FeCoPt-1 | Down | -14.417 | Down | -14.600 | Down | -47.978 |
|  | Net spin | 0.298 | Net spin | 0.045 | Net spin | -0.068 |
|  | Up | 14.680 | Up | 24.379 | Up | 38.146 |
| FeCoPt-2 | Down | -14.379 | Down | -24.223 | Down | -38.210 |
|  | Net spin | 0.301 | Net spin | 0.156 | Net spin | -0.064 |
|  | Up | 9.767 | Up | 34.208 | Up | 33.312 |
| FeCoPt-3 | Down | -9.576 | Down | -33.883 | Down | -33.369 |
|  | Net spin | 0.191 | Net spin | 0.325 | Net spin | -0.057 |
|  | Up | 9.748 | Up | 39.074 | Up | 28.502 |
| FeCoPt-4 | Down | -9.559 | Down | -38.681 | Down | -28.573 |
|  | Net spin | 0.189 | Net spin | 0.393 | Net spin | -0.071 |
|  | Up | 11.579 | Up | 50.913 | Up | 28.326 |
| FeCoPt-5 | Down | -11.376 | Down | -50.683 | Down | -28.462 |
|  | Net spin | 0.203 | Net spin | 0.230 | Net spin | -0.136 |

**Table S6.** The d-band centers of different samples.

| **Sample** | **Fe (eV)** | **Co (eV)** | **Pt (eV)** |
| --- | --- | --- | --- |
| FeCoPt-2 | -0.498 | -0.765 | -2.545 |
| FeCoPt-2-(1) | -0.518 | -0.783 | -2.516 |
| FeCoPt-2-(2) | -0.561 | -0.847 | -2.599 |
| FeCoPt-2-(3) | -0.237 | -0.516 | -2.140 |

**Table S7.** The net spins of different samples.

| **Sample** | **Net spin (μ_B_)** | | |
| --- | --- | --- | --- |
|  | **Fe** | **Co** | **Pt** |
| FeCoPt-2 | 0.301 | 0.156 | -0.064 |
| FeCoPt-2-(1) | 0.300 | 0.165 | -0.056 |
| FeCoPt-2-(2) | 0.284 | 0.110 | -0.065 |
| FeCoPt-2-(3) | 0.240 | 0.195 | -0.058 |

**Table S8.** The centers of the d-bands for spin-up and spin-down states of different samples.

| **Sample** | **Fe up (eV)** | **Fe down (eV)** | **Co up (eV)** | **Co down (eV)** | **Pt up (eV)** | **Pt down (eV)** |
| --- | --- | --- | --- | --- | --- | --- |
| FeCoPt-1 | -2.419 | 0.391 | -1.457 | -0.763 | -2.630 | -2.446 |
| FeCoPt-2 | -2.442 | 0.447 | -1.594 | -0.327 | -2.558 | -2.303 |
| FeCoPt-3 | -2.168 | 0.608 | -1.990 | -0.135 | -2.490 | -2.179 |
| FeCoPt-4 | -2.014 | 0.694 | -1.972 | -0.122 | -2.413 | -2.099 |
| FeCoPt-5 | 0.823 | 3.160 | 0.496 | 2.150 | 0.610 | 0.913 |

**Table S9.** The splitting of the spin-up and spin-down d-band centers of different samples.

| **Sample** | **Fe (eV)** | **Co (eV)** | **Pt (eV)** |
| --- | --- | --- | --- |
| FeCoPt-1 | 2.810 | 0.694 | 0.184 |
| FeCoPt-2 | 2.889 | 1.267 | 0.255 |
| FeCoPt-3 | 2.776 | 1.855 | 0.311 |
| FeCoPt-4 | 2.708 | 1.850 | 0.303 |

**Table S10.** The correspondence between experimental and theoretical magnetic moment.

| **Magnetic field (Oe)** | **Experimental magnetic moment (μ_B_)** | **Theoretical magnetic moment (μ_B_)** | | |
| --- | --- | --- | --- | --- |
|  |  | **Fe** | **Co** | **Pt** |
| 3500 | 6.42 | 1.12 | 0.84 | 0 |
| 4000 | 7.61 | 1.36 | 1.02 | 0 |
| 4500 | 8.83 | 1.56 | 1.17 | 0 |
| 8000 | 15.49 | 2.76 | 2.07 | 0 |
| Saturation | 22.56 | 4.00 | 3.00 | 0 |

1. ^*^ Corresponding authors. E-mail: jialongliu@buct.edu.cn (Jialong Liu); wangwei@mail.buct.edu.cn (Wei Wang).
